# Exploring barriers to accessing healthcare services for older indigenous people in the Chittagong Hill Tract, Bangladesh

**DOI:** 10.3934/publichealth.2023047

**Published:** 2023-08-10

**Authors:** Md. Sohrab Hossen, Md. Salman Sohel, Gazi Abu Horaira, Md Aminul Haque Laskor, Asia Binta Amanat Sumi, Srima Chowdhury, Sima Aktar, Md. Khaled Sifullah, Md. Fouad Hossain Sarker

**Affiliations:** 1 James P Grant School of Public Health, BRAC University, Dhaka, Bangladesh; 2 Department of Development Studies, Daffodil International University, Dhaka–1216, Bangladesh; 3 Ghazali Shafie Graduate School of Government, University Utara Malaysia, Malaysia; 4 Department of Geography and Environment, Shahjalal University of Science and Technology, Sylhet, Bangladesh; 5 Department of International Business, University of Dhaka, Dhaka, Bangladesh; 6 Faculty of Business Administration, University of Chittagong, Chittagong, Bangladesh; 7 Eden Mahila College, University of Dhaka, Dhaka, Bangladesh

**Keywords:** healthcare access, older, indigenous, Chittagong Hill Tracts (CHT), Bangladesh

## Abstract

We aim to investigate the obstacles faced by elderly indigenous individuals in the Chittagong Hill Tracts, Bangladesh when accessing healthcare services. A qualitative research approach was utilized, and data collection was carried out in three distinct regions of the aforementioned area. A total of 30 in-depth, semi-structured interviews and participant observations were conducted to achieve the research objectives. Thematic analysis utilizing both a deductive and inductive approach was employed to analyze the data. The Granheim method and Nvivo-12 software were utilized to process, analyze and code the data. The study's findings indicate that a lack of knowledge about healthcare needs, geographical barriers, poor financial conditions, higher cost of medical services, scarcity of hospitals nearby and communication barriers all contribute to inadequate access to healthcare services. By recognizing the factors that impede access to healthcare services in this region, this study offers valuable insight for policymakers and healthcare providers on how to enhance healthcare services for the indigenous population, especially the elderly. Furthermore, the government can adopt a more efficient approach to include these elderly individuals in various social safety net programs.

## 1. Introduction

Indigenous Peoples are native inhabitants of specific geographic areas who have often experienced colonization and domination by external cultures [1],[2]. They possess unique cultural identities and ancestral ties to the lands they currently inhabit, historically occupied, or were displaced from [3]. Globally, Indigenous Peoples, numbering 476 million across 5,000 cultures in 90 countries, constitute only 6% of the total population but account for approximately 19% of those living in extreme poverty [4]–[6]. Consequently, they face numerous social, economic and environmental challenges that adversely affect their health and well-being, resulting in a life expectancy up to 20 years shorter than non-indigenous individuals [7]–[10].

Indigenous communities worldwide encounter common factors contributing to health inequalities and limited healthcare access, such as residing in remote areas, social isolation, financial deprivation, linguistic obstacles and lack of nearby healthcare facilities [11]–[15]. In Asia, where indigenous communities represent 70% of the global indigenous population, additional barriers include neglect by national governments and culturally inappropriate healthcare services, resulting in poor health outcomes, high mortality rates, malnutrition and a disproportionate burden of poverty-related diseases [16]–[20].

Bangladesh is home to millions of climate induced displaced people [21]–[25], migrants [26]–[28] refugee [29] and huge indigenous population [30]. Among South Asian nations, Bangladesh has 54 dispersed indigenous communities, with 1.65 million indigenous people residing in both plains and hills [31],[32]. The Chittagong Hill Tracts (CHT) region, covering 10% of the country's land area, harbors 13 distinct indigenous groups making it approximately 55.76% of the total indigenous population, with the largest populations belonging to the Chakma and Marma communities [32]–[34]. Each village within this region has its distinct set of customs, encompassing unique languages, clothing styles and economic pursuits [35],[36].

The CHT region is characterized by geographical remoteness, low-income levels, limited employment opportunities, high conflict rates, land-grabbing practices, extreme poverty, inadequate healthcare facilities, limited access to clean water and sanitation, subpar educational resources and deficient infrastructure development [31],[37]. The poverty rate among the indigenous people is greater than the country's other regions [34]. Despite these challenges, the healthcare needs of the indigenous population in the CHT region receive insufficient attention, highlighting the urgent need for healthcare equity and universal health coverage in Bangladesh [18],[38],[39].

Due to the susceptibility of Indigenous communities to healthcare challenges, the elderly Indigenous population appears to be particularly vulnerable. This is made worse by the fact that a sizable amount of Bangladesh's aged care requirements is still unmet or ignored [40]. The older population in Bangladesh encounters numerous obstacles when seeking healthcare services, including insufficient healthcare infrastructure, limited financial resources, socioeconomic instability and neglect [41],[42].

Through a comprehensive literature review, we found several relevant studies on healthcare challenges faced by indigenous populations in Bangladesh. These included research by Saha et al. (2022) [43], on the prevalence of self-medication and its impact among indigenous communities in the Chittagong hill track area, Rahman et al. (2021) [44], on the health status and quality of life of older indigenous individuals in the Sylhet region, studies on the challenges of maternal healthcare access for indigenous women [45] and research by Lewis and Myhra (2018) on healthcare disparities in rural areas affecting indigenous populations [46].

However, to the best of our knowledge, no research has investigated the healthcare access challenges encountered by the Indigenous older population residing in the Chittagong Hill Tracts region of Bangladesh. Consequently, we aim to conduct a qualitative, in-depth analysis of the barriers that impede Indigenous older people's access to healthcare services in this region. Thus, the primary objective of this study is to gain an understanding of the underlying issues that hinder healthcare access for this vulnerable population, which is essential in developing effective and culturally appropriate healthcare policies and interventions that can promote better health outcomes and improve the overall well-being of Indigenous peoples in the region. Moreover, this study aligns with the SDG 3 objective, which seeks to ensure healthy lives and well-being for all, regardless of age. Consequently, this research has the potential to narrow the existing knowledge gap on Indigenous health by bringing attention to the distinctive challenges faced by this vulnerable group. The results of the study can guide healthcare policy and practice, improving health outcomes for the elder Indigenous population.

## 2. Materials and methods

### 2.1. Ethics approval

Ethical approval (Protocol No. Ethics/ salman7/2022) has been obtained from the Institutional Ethical Review Board affiliated with the Faculty of Humanities and Social Science at Daffodil International University, located in Dhaka-1212, Bangladesh.

### 2.2. Study area and location

**Figure 1.**
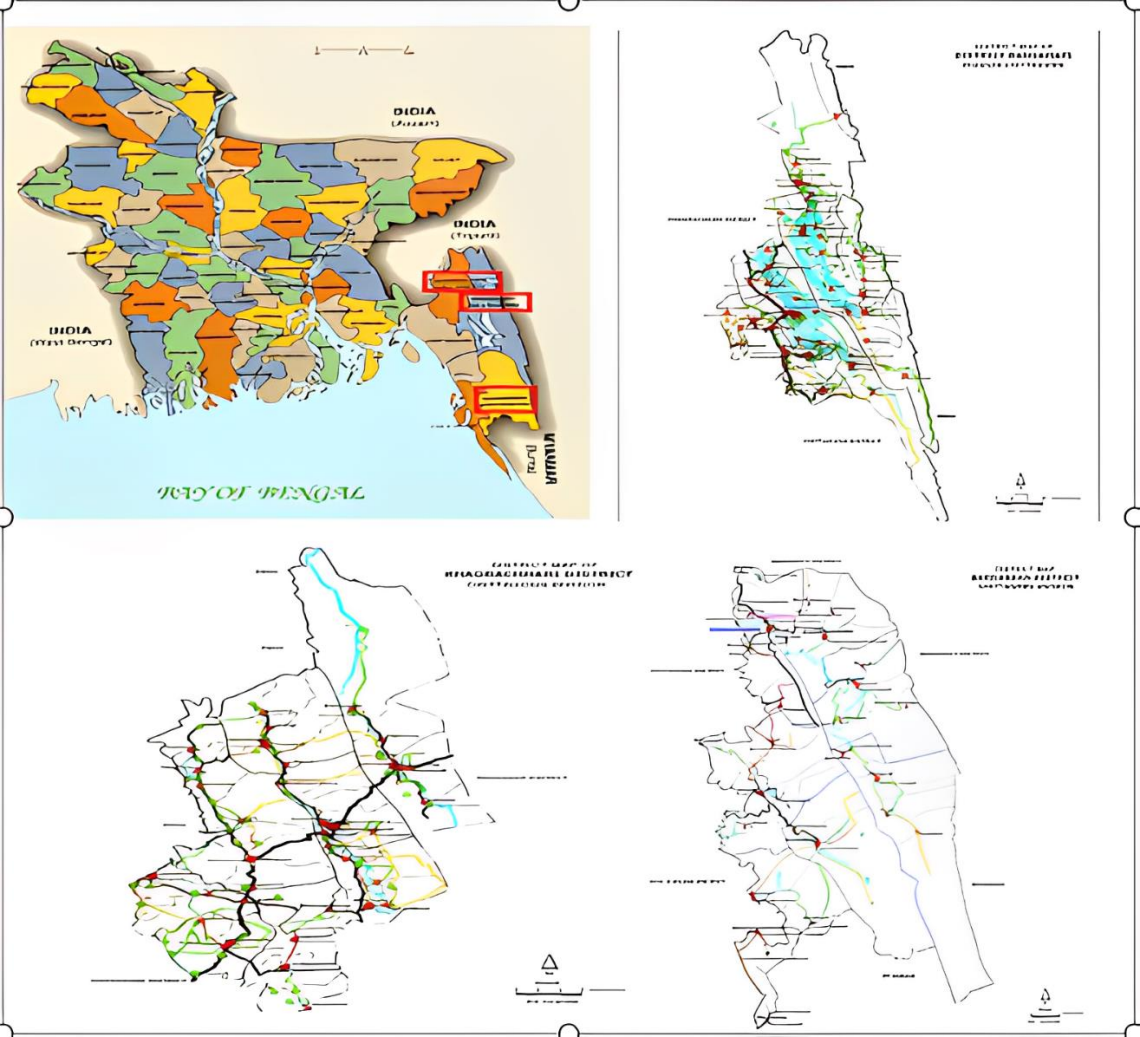
Location of the study area.

The Chittagong Hill Tracts (CHT) encompass three hilly districts, namely Rangamati, Khagrachari and Bandarban, which collectively cover 10% of Bangladesh's total land area. It shares its borders with the Indian state of Tripura to the north, the district of Bandarban to the south, the state of Mizoram and the Myanmar state of Chin to the east, and the districts of Khagrachari and Chittagong to the west. The region has a subtropical climate, with an average annual temperature range of 10°C to 35°C and a mean rainfall of 2500 mm [47]. The study area is depicted in the Figure 1.

### 2.3. Study design and sample size

We used a cross-sectional qualitative research approach for this study as it is increasingly used by applied health researchers to give voice to vulnerable populations like indigenous communities [48],[49]. Crouch & McKenzie, (2006) recommended having less than 20 respondents for qualitative research, as it helps establish intimate connections and improve communication [50]. For qualitative research, Sandelowski, (1995) advised using a sample size of 10 involving homogeneous individuals [51]. However, Green, J. and Thorogood, (2004) noted that most qualitative researchers use 20 or more participants [52]. For our study, we used purposive sampling to select 30 participants for face-to-face in-depth interviews to collect qualitative data.

### 2.4. Data collection and instruments

We conducted research on the Chittagong Hill Tracts (CHT) in Bangladesh, which consists of three hill districts: Rangamati, Khagrachari and Bandarban. Ten in-depth interviews were collected from Rangamati, ten from Khagrachari and ten from the Bandarban area. To gain a better understanding of the practical challenges related to our research topic, we conducted semi-structured interviews with participants. These interviews allowed participants to provide detailed and comprehensive responses based on their knowledge and experience [53]. Moreover, semi-structured interviews are effective in obtaining accurate and detailed information about the research topic. We collected our data between February and March 2022, and at the start of the data collection, we took five interview samples from the study's field sites. After the first interviews, the interview guide was refined and this process of questionnaire creation is widely used in qualitative research [54]. The interviews were recorded using mobile devices and their duration ranged from 15 to 22 minutes. In cases where respondents were unwilling to speak while being recorded, written responses were taken. We also observed the respondents' attitudes, expressions, tone and working atmosphere during the interviews. Subsequently, the recorded interviews were transcribed with great care and accuracy, and the researchers double-checked them for correctness. All respondents provided oral consent to participate in the interviews and they were informed that participation was optional. Furthermore, we assured them that their study materials would not be shared for any purpose in the future.

### 2.5. Data analysis procedures and approaches of measure and coding

The researchers utilized the Nvivo-12 software to effectively organize and classify the interview transcripts, resulting in improved management of crucial information and heightened coding quality, thereby strengthening the reliability and validity of the findings [55]–[58]. The data were collected and categorized by profession in a separate file, which was later compiled and triangulated based on its nature, type and features [56]. The researchers employed a multiple triangulation technique to verify the quality, validity and dependability of the data [59],[60]. To analyze the data, the researchers utilized a hybrid approach that integrated a data-driven inductive method and a deductive approach, with thematic analysis being the chosen method [61],[62].

The Granheim and Lundman technique [63] was utilized by the authors to analyze, process and code the data, which is a commonly employed method in social science and public health research. The Graneheim and Lundman approach to content analysis served as the fundamental framework for data analysis in this study, allowing for comparisons and contrasts within and between various data sets. Qualitative content analysis, as described by Graneheim and Lundman, focuses on examining a text's explicit or manifest content while also providing interpretations of its “latent content.” The specific data analysis techniques utilized in this study are thoroughly outlined in Table 1.

As noted by Yin (2011), it is crucial for researchers to carefully select quotations or excerpts that strengthen their interpretations and justifications [64]. However, researchers must also make informed decisions about the selection, appropriateness and accuracy of these quotations or excerpts, as there are no established guidelines for their number or length [65]. The decision to include such quotations or excerpts is dependent upon how researchers interpret and contextualize the theme or sub-theme within their research. These illustrative excerpts or verbatim accounts can be powerful indicators of the researcher's comprehension of the population's viewpoints. In this study, the authors have taken great care in selecting meaningful quotations that accurately reflect the narrative situation and support the presented results.

**Table 1.** Thematic data analysis using the Granheim and Lundman approach.

**Steps**	**Description**
**1. Transcription of Interviews**	The first step involved recording the interviews and carefully listening to the recordings several times to fully understand their contents. Then, the interviews were transcribed for further analysis.
**2. Unit for the formation of meaning analysis**	The results of each interview have been examined separately. Making primary codes by removing meaningful units
**3. Comprehensive sorting of similar codes**	This step involved organizing primary codes with similar characteristics into broader categories for a more comprehensive analysis.
**4. Comparison of codes and establishment of subcategories**	On the other hand, through the examination of all codes and data, similarities and differences were recognized, leading to the development of categories and subcategories.
**5. Comparing subcategories and establishing primary categories**	After the initial interviews, an initial set of codes, categories and subcategories were generated, and the resulting emerging codes were considered as the outcome of the content analysis methodology. To ensure the accuracy and consistency of the findings, two independent researchers examined the category data.

**Figure 2.**
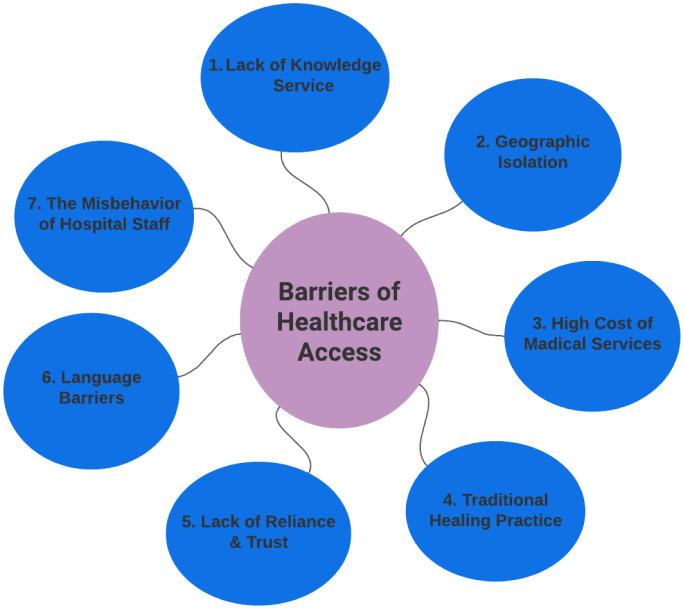
Barriers to healthcare access.

To analyze the qualitative data, the NVivo-12 software was utilized, resulting in the identification of seven major themes. The theme “lack of knowledge about healthcare services” received the highest reference codes, whereas the “misbehavior of hospital staff” theme had the lowest reference codes using the NVivo-12 software. Figure 2 was developed by the authors utilizing field data, and the thematic reference coding produced by the NVivo-12 software is shown in Table 2.

## 3. Results

Table 3 presents a summary of the demographic characteristics of the participants who took part in this research study. The age range of the participants was from 50 to 80 years old, with a total of 30 participants, consisting of 15 males and 15 females. The majority of the participants had no formal education, and only a few had completed primary school education. Furthermore, nearly all of the participants were married, whereas 11 of them were widowed. Notably, people from the Chakma and Tripura ethnic groups dominated the research sample.

Health is wealth and crucial for development, but providing it in underdeveloped nations is very challenging. Especially healthcare access for older people living in the Chattogram Hill track region is vital due to the numerous challenges and problems that they face. Based on our Nvivo analysis, the major barriers in this region are lack of knowledge service; geographic isolation; high cost of medical services; lack of reliance and trust; language barriers and the misbehavior of hospital staff. Additionally, older people in this region often suffer from complex medical conditions and chronic illnesses, further exacerbating the need for accessible and high-quality healthcare services. In this case, figure 2 presents the barriers to accessing healthcare services for older indigenous people in the Chittagong Hill Tracts.

**Table 2.** Defined themes derived from the thematic analysis.

**Theme**	**Reference code**	**Descriptive coding**
Lack of Knowledge about Healthcare Services	176	*“Once I could go to my neighbors, but because of my illness I cannot walk nowadays.”*
Geographic Isolation	167	*“Many people in our community are unaware of healthcare services, particularly those who are not part of the Chakma community.”*
High Cost of Medical Services	154	*“Government hospitals are more affordable in contrast to private hospitals. Some people visit the government hospital due to the low cost.”*
Traditional Healing Practice	150	*“For the past ten years, I've been taking traditional medicine. Traditional medicine does not provide good results, but people take this.”*
Lack of Reliance and Trust	139	*“I went to the clinic for pain in my leg, but it did not heal. Now there is no more faith. How many more years I will live? I will stay like this. I will die when the time comes.”*
Language Barrier	138	*“People don't understand the Pahari (Indigenous) language. We don't know what to say, what not to say. That's why I don't want to go to the hospital.”*
The Misbehavior of Hospital Staff	132	*“I often find myself struggling to understand the medical instructions given to me by the doctors and staff, and sometimes they become impatient and frustrated with me, mostly when there is a crowd. It is also true that they assume I can't understand them because of my age or background.”*

### 3.1. Lack of knowledge of healthcare services

The study respondents asserted that although there is a shortage of healthcare facilities in the CHT, they cannot even utilize the opportunity because of a lack of healthcare knowledge. The cause behind this is that they are uneducated and cannot recognize their health needs. In addition to that, being older people, as they live in remote areas, their exposure to healthcare information and awareness campaigns is limited. Also, these people do not have digital literacy to access information. Thus, their sources of information are their immediate family members or neighbors. A respondent stated,

*“My younger son once said to me that healthcare facilities are available in the nearby town, but I don't know how to get there or what services are available. I don't have access to information about healthcare services. At this age, I feel like I am missing out on opportunities to take care of my health, but I don't know where to start.” P#17*

So, there is no formal way of knowing health care services for them. Their source of information is basically gossiping among the neighbors. While having this kind of informal discussion, they discuss health care services or with someone who had gone to a hospital to take health services. However, because of the mobility issue of older people, communication with neighbors or having some informal discussion as well is rare and difficult for most older people. For those older people who have no immediate family, and are suffering from a chronological illness which hinders their mobility, their access to information about health care is censurably in the worst situation. Also, the remote areas of CHT are not really appropriate for these older people to travel. As participants asserted,

*“Our knowledge of healthcare services comes through conversations among the villagers, our neighbors and even our children.” P#1,12,16,30*

In addition to that, our respondent from the Chakma group, which is the largest ethnic group and one of the most influential groups in CHT, informed us that they have a Chakma circle there. People occasionally know about healthcare services through this circle's group discussion. However, the representation of female older people is considerably low in this discussion. Thus, they lag in terms of health care awareness than their male counterparts. Also, this kind of circle is not available in other communities as they are fewer in number. So, the majority of them were found unaware of the health care needs and services. As two of the older male respondents from the Chakma community said,

*“Most of the people in our community are from the Chakma community and we have our community circles. Our Chakma circle informed us about health care services. However, many people in our community are unaware of healthcare services, particularly those who are not part of the Chakma community.” P#10,14*

**Table 3.** Distribution of demographic profile of the interviewees.

**Category**	**Variable**	**N**
**Gender**	Men	15
	Women	15
**Age**	50–60	2
	60–70	18
	70–80	10
**Education**	Illiterate	27
	Primary school	3
**Marital Status**	Married	18
	Widow	11
	Unmarried	1
**Ethnicity**	Chakma	10
	Marma	5
	Tripura	10
	Boom	5
**Place of Residence**	Khagrachari	10
	Rangamati	10
	Bandarban	10
**Occupation**	Daily labor	7
	Agriculture	3
	Housewife	14
	Retired	1
	Jum cultivation	4
	Bunon artist	1

Note: Source: Field Survey.

### 3.2. Geographic isolation

The Chittagong Hill Tracts differs from the rest of Bangladesh due to its mountainous, rough terrain, dense forests, lakes and falls. Due to these kinds of exceptional features, there are a few medical centers. Also, the population density is less in this area. So, there is a considerable distance from one house to another. The same goes for the health care centers as well. Since there are not as many health centers, their distance from the houses is also considerable considering the geographical location of this area. On top of that, our study participants, the older people, live mostly alone as it is common that their children live separately after marriage. So, because of age, they alone cannot go to the medical centers because of the long distance. They need to seek others' help which they cannot always get because of the unavailability of the immediate family nearby and indigenous people are mostly busy with their work.

*“I have lived in the Chittagong Hill Tracts all my life, and as I grow older, it becomes more challenging to access healthcare services. Our house is a long way from the healthcare center. The range of travel distance is roughly 10 to 15 kilometers. It takes a lot of time to go to the healthcare center.” R#23*

*“I cannot travel alone due to my age. My children have moved away after marriage, and I cannot always rely on others for help.” R#26*

Additionally, with poor infrastructure, limited transportation options and difficult terrain, accessing healthcare services is a constant struggle for old people. During the daytime, they may be able to catch a local bus, but at night, they are left with no option but to rent a private vehicle, which is often beyond their financial means. The reasons for the lack of availability of vehicles in the areas are due to the sparse population, low demand, steep terrain and poor financial condition. Thus, this limited number of vehicles in these areas, combined with high fuel costs as there are no fuel stations nearby, makes transportation a significant expense for this vulnerable population. Furthermore, extreme weather events such as floods, excessive rainfall, landslides and thunderstorms make accessing healthcare even more difficult. As some of our participants said,

*“When we are sick, we must face several difficulties to go to the hospital. There are several reasons behind it including lack of transportation and improper infrastructure. Additionally, when the entire region is under water due to a flood, finding transportation is really challenging. Also at night, there are no vehicles. It's like we are forgotten by the rest of the world.” R#9, 16*

### 3.3. High cost of medical services

Our study participant stated that in the Chittagong Hill Tracts area, because of the sparse population, the hilly terrain, the high cost of transportation and the lack of qualified doctors nearby medical services are quite expensive. The financial status of inhabitants in The Chittagong Hill Tracts is extremely poor [34],[66]. Additionally, as mentioned earlier, in their culture, it is common that the children have separated after marriage and their parents become lonely. So, the situation for old people who cannot work anymore or have limited income is challenging for them to access healthcare services that require payment. The cost of healthcare services leads to Indigenous older people not seeking care when they need it, which can result in worsening health outcomes. The majority of the participants reported that this applied to them.

*“The cost of healthcare is extremely high. The cost of high blood pressure treatment is between BDT 2000 and 3000. Sometimes the Rangamati Hospital is unable to provide services, that time one must go to Chittagong which is not possible for everyone. In that case, the cost is alarming for us. For one visit, it takes a minimum of BDT 10000 including the test.” R#1, 2, 12, 30*

There are both public and private healthcare facilities in Chittagong Hill Tracts. Government hospitals are less expensive than other private hospitals. However, the service in the government hospital is comparatively inferior. People who are struggling financially visit government hospitals. As one of the respondents said,

*“Government hospitals are more affordable in contrast to private hospitals. Due to the affordable prices, several individuals use the government hospital.” R#15*

As a result of their limited financial resources, elderly people frequently chose to obtain medications from local drug stores rather than seeking medical treatment at hospitals when they fell ill. This choice was driven by the necessity to manage their financial constraints effectively. By opting for local drug stores, they were able to access affordable medication without incurring the significant costs associated with hospital visits.

*“If I'm sick, I go to the local drug store and bring medicine at low cost. Going to hospitals means spending lots of money” R#28*

### 3.4. Traditional healing practice

The World Health Organization defines traditional medicine as the totality of knowledge, skills and practices based on theories, beliefs and experiences that are inherent indigenous to different cultures, whether they can be explained, and used in the maintenance of health as well as in the prevention, diagnosis, improvement, or treatment of physical and mental illness [67]. Most of Bangladesh's traditional medical practices are based on locally accessible substances, cultural customs and religious rites. Ayurvedic and Unani systems that make use of scientifically supported pharmaceutical techniques and technology also play a significant role in traditional medicine [68]. In our study, we found many indigenous communities have their traditional healing practices, and the older indigenous people were found more reliance on this practice because they believe their ancestors used it and got cures quite effectively. This results in indigenous people not seeking Western medical care, even in cases where it may be necessary. As one of the respondents said,

*“For the past ten years, I've been taking traditional medicine. Traditional medicine does not provide good results, but people take this.” R#1*

### 3.5. Lack of reliance and trust

According to the BBS (2011), the average literacy rate in the Chittagong Hill Tracts is 43.9 per cent, which is lower than the national average of 51.8 per cent [69]. This literacy percentage demonstrates that most people in Chittagong Hill Tracts are uneducated. This rate is higher among the older indigenous people. Due to a lack of knowledge, they believe modern medicine does not work properly. They do not go back to the doctor if they do not get a good result by taking medicine. As two of the respondents said,

*“I went to the clinic for pain in my leg, but it did not heal. Now there is no more faith. How many more years I will live? I will stay like this. I will die when the time comes.” R#28*

### 3.6. Language barrier

Bangladesh has a very diverse population in terms of race, religion, language and culture. A remarkable 98% of its residents identify as being from Bangladesh and speak the native tongue, Bangla. According to a recent study by the International Mother Language Institute of Bangladesh, in Chittagong Hill Tracts, there are 41 ethnic languages [69]. In Chittagong Hill Tracts, indigenous people, particularly the old people, do not speak or understand the Bengali language. It limits their ability to communicate their healthcare needs properly with the healthcare professionals or understand the information provided to them by healthcare providers which makes it challenging to get proper healthcare. Furthermore, it affects their willingness to seek healthcare services.

*“People don't understand the Pahari(Indigenous)language. We don't know what to say, what not to say. That's why I don't want to go to the hospital” R#26*

### 3.7. The misbehavior of hospital staff

The respondents said the government hospitals in the city remain crowdy most of the time. So, even though they somehow manage to reach there, the staff do not properly treat them because of a lack of proper communication between the medical staff and older patients. Older people have difficulty understanding complex medical instructions, and because of this doctors and staff become frustrated with their inability to communicate effectively. Sometimes the doctors even do misbehave with them when the older people fail to explain the illness to the doctors. Furthermore, the participants also pointed out another reason for misbehavior they think doctors and staff hold unconscious biases towards older patients, assuming that they are unable to understand or communicate effectively. According to a respondent,

*“I often find myself struggling to understand the medical instructions given to me by the doctors and staff and sometimes they become impatient and frustrated with me, mostly when there is a crowd.” R#13*

*“It is also true that they assume I can't understand them because of my age or background” R#21*

## 4. Discussion

Our study found several factors that have a major impact on indigenous old people's healthcare access. Thus, this study adds significant information to our understanding of the factors that act as barriers to accessing healthcare services for the old indigenous people living in the CHT region. Data revealed that socio-economic positions, discrimination, language barriers and geographical isolation inhibit the old indigenous populations to access healthcare. Among these factors, some others like limited mobility, language barriers and prohibitive costs, living separately from children make the scenario worse. As a result, they are avoiding seeking medical attention until critical situations arise. These challenges ultimately negatively impact the overall health and well-being of the elderly population.

First, the indigenous old people in the CHT regions cannot utilize the limited opportunities for health care in the region because of the lack of knowledge about health care needs. This is because, among old people, illiteracy is high which limits their capacity to understand health issues. Also, as they live in remote areas, they have limited exposure to healthcare information and awareness campaigns. Because, as they are older, and have mobility issues, they cannot even go to the place where campaigning happens. Thus, they primarily rely on their immediate family and neighbors for healthcare information. However, there is a question of the reliability of that information, as all of them share almost the same socio-economic condition. It impacts their healthcare-seeking behaviors as we found some of the participants believed the illness comes naturally and cures on its own, and such belief demotivates them to seek healthcare. Because of this, preventive care, early detection and treatment options to maintain good health and well-being are extremely difficult for them. Also, as they do not know about their health risks, how to manage chronic conditions or where to go for medical care, has led to delayed treatment, worsening of conditions and higher healthcare costs. Similar results were discovered by Akter et al., (2020), and this study mentioned that lack of knowledge about healthcare services among indigenous women leads to the worst healthcare-seeking behavior in Bangladesh decreasing the value of treatment and efficiency of treatment [45]. The UN report also highlights the limited healthcare access experienced by indigenous populations worldwide due to their lack of knowledge about healthcare needs in Central Africa [18].

Second, our study found that geographical remoteness poses a significant challenge to healthcare access among the elderly in the CHT region, characterized by mountainous terrain, dense forests, lakes and waterfalls. Limited availability of healthcare centers located far from residential areas, coupled with poor infrastructure, limited transportation options and difficult terrain, further hinders access to healthcare services. This scenario is not unique to the CHT region but is shared by many indigenous communities worldwide [18]. This remoteness is today a major threat to the Greenlandic healthcare system because of lack of staff on all professional levels and their rapid turnover, especially for small towns and remote villages [70].

Next, the cost of seeking healthcare has been the most challenging issue for seeking healthcare, according to the participants. Overall, the out-of-pocket expenditure on health care is very high in Bangladesh [71]. On average, Bangladeshi citizens must pay 63.3% of their total treatment costs out of their own pockets [72]. A study found that about 26% of households incurred catastrophic health expenditures in Bangladesh [73]. The problem is simply worse in the CHT region because of its geographical structure which results in substantial transportation costs, which pose a financial burden for the vulnerable older population. This added expense, along with healthcare costs, leads to a situation where elderly individuals only seek healthcare when their condition becomes critical and no other options are available. These findings align with those who found that location is one of the main reasons for the inequity in healthcare services which occurs between urban-area people and indigenous people [11]. A similar result was found by Afsana, (2004), and in her study, she asserted that in rural areas of Bangladesh, women with obstetric problems are reluctant to seek medical care for a variety of reasons, including the exorbitant expense [74]. It is evident in Nepal where 43 per cent of indigenous women have reported that they are unable to receive health care services due to lack of money [18]. Another study by A. Islam, (2014) revealed that the cost of medicines and laboratory testing, as well as certain extra hidden charges, are ultimately borne by patients even though basic healthcare services in public hospitals and other institutions are meant to be provided without charge [75]. The majority of publicly financed healthcare services are severely out of reach for the poor and the disadvantaged due to these expenses.

In addition, our study identified a lack of trust in healthcare as significant factor influencing the healthcare-seeking behavior of older indigenous individuals. It was observed that if they did not experience positive outcomes from medication, they would refrain from returning to the doctor, perceiving the medicines as ineffective. This mistrust and reluctance to seek medical care can be attributed to the historical discrimination and trauma experienced by indigenous people in the CHT region of Bangladesh [76], impacting their trust in healthcare providers and institutions.

Furthermore, the communication gap also brings forth new challenges. Due to the language barrier, the old people cannot express their health problems to the service providers nor do they understand the instruction of the medical staff properly. As a result, participants have often complained about employees acting inappropriately towards them. Participants also reported that they think doctors and staff hold unconscious biases towards older patients, assuming that they are unable to understand or communicate effectively is one of the reasons they misbehave. These biases can lead to poor treatment and inadequate care, which can be particularly harmful to older people, who may have complex medical needs. A study in South Africa found that because of the harsh treatment from the nurses, the indigenous women shied away from delivery in hospitals [77]. Apart from this, language barriers to accessing health care was also found in a previous study [45]. The study also found that Indigenous women from Chittagong's most isolated highland regions were unable to communicate fluently in Bengali, especially when it came to medical terms. Because of these factors, we found some of them rely on their traditional healing practice, even if it does not work, rather vising healthcare centers. The traditional healing practice was also reported by the other study [43],[78]. The study concluded that culture, inadequate education, ignorance of modern medical care, underdeveloped transportation and communication systems and general backwardness from modern society forced indigenous people in Bangladesh to use their traditional medical systems. In addition, healthcare providers often do not recognize this as a valid form of treatment. As a result, older indigenous people may feel uncomfortable seeking healthcare services or may receive inadequate care.

## 5. Policy recommendations

Bangladesh consistently falls behind other countries in healthcare rankings, particularly when it comes to the indigenous healthcare system, because of its inadequate healthcare system and dense population. Due to a lack of awareness, healthcare is not a major concern for indigenous people. To raise awareness of healthcare among indigenous people, the government and non-government organizations must work together. The awareness-building efforts should be engaging local resources like community leaders so that engaging the community becomes easier and the acceptability of the initiatives increase. Also, using visual aids and simple language for example pictures and diagrams, and simple language is required to convey healthcare information because the majority of old indigenous people are illiterate or did not have access to formal education. People in Chittagong Hill tracts avoid visiting hospitals because of their financial situations, especially the elderly, and instead rely on local drug stores for medical care. Their healthcare cost seems higher because of the higher transportation costs. So, the government should be providing subsidies for these vulnerable people not only in government hospitals but also in private hospitals as well, so that they can access healthcare at a low-cost whichever facility is around them. Apart from that, incorporating these old people into different social safety net programs on a larger scale would be a more effective support for unemployed indigenous people. The government need an authentic database to identify the unemployed and older lonely people to support them in need and should take the necessary steps to increase the healthcare center and government transport in that area. To treat minor illnesses, some tribal people in the Chittagong Hill Tracts use traditional medicine. Because they believe their predecessors practiced traditional medicine and recovered, they continue to practice it even though it frequently fails. Some of them also consider that modern medicines do not function properly. They believe this is because they do not know enough about it. In this situation, the government should concentrate on the expansion of education. Increasing the number of schools, colleges and universities there as well as encouraging the children of tribes to get an education. In addition, hospital accommodations are limited and of poor quality, notably in government hospitals. Private hospitals occasionally offer accommodation, although the cost is significantly high, and some patients frequently receive inferior treatment from the medical staff in Chittagong Hill Tracts. The government should pay heed to this issue by strictly monitoring the activities of the staff and motivating them to provide services to these vulnerable people. Indigenous inhabitants of the Chittagong Hill Tracts speak their native language; hence they are unable to understand Bengali. They are consequently unable to effectively convey their medical issues to a Bengali doctor. In this scenario, the government should ensure the presence of doctors who are proficient in the native language of the communities being served in each hospital or at least assistants belonging to the respective communities, in addition to Bengali doctors. By implementing these steps, healthcare in the Chittagong Hill tract will be more accessible and encourage more inhabitants to utilize it. Additionally, there will be a significant reduction in health-related issues.

## 6. Conclusion

Indigenous people of CHT are vulnerable due to their socio-economic condition and thus, as in many things, are deprived of access to healthcare services. In this context, the old people are the most vulnerable class since most individuals in their sixties run the danger of having their incomes decline, which for some may lead to poverty which would then restrict their access to healthcare [79], which in turn shrink their access to healthcare. Hence, the purpose of the study was to assess the existing barriers that impede the old indigenous people in CHT regions to access healthcare services. The study used a cross-sectional qualitative research approach to get an in-depth idea of healthcare-seeking behavior and barriers to accessing healthcare services in Chittagong Hill Tracts. We thematically analyzed the interviewed data using Nvivo-12 software. By analyzing the data, this study concludes that people lack access to health services because of a lack of knowledge about healthcare needs, geographical barriers, poor financial conditions, higher cost of medical services, a scarcity of hospitals nearby and communication barriers. The government and non-government organizations must take collaborative action to increase indigenous peoples' awareness of healthcare and find a more efficient way to incorporate these old people in various social safety net programs. The government also ought to pay attention to this region's literacy rate. Establishing more schools, colleges and universities, may encourage tribe youth to pursue education who will be able to be aware of their other family members like the old people in the family.

It is important to note that this article still has some limitations. This study uses just a qualitative methodology, and deals with a smaller population. Thus, we are unable to generalize our results. Furthermore, the data provided insightful information on the elderly indigenous people of CHT's susceptibility to needing medical attention. Future research, however, may use mixed-technique approaches or huge quantitative samples. Identifying the most critical healthcare requirements of these poor people and figuring out how to include them in social welfare initiatives may be the next research step. Since the findings in this study can represent a base for the government, policymakers, aid organizations and associated agencies, they should adopt this study to comprehend the existing scenario of healthcare services in Chittagong Hill tracts.

## Use of AI tools declaration

The authors declare they have not used Artificial Intelligence (AI) tools in the creation of this article.
